# Anthocephalus indicus and Terminalia arjuna Combination Ameliorates Hematological and Histopathological Changes in a Rat Model of Metabolic Syndrome

**DOI:** 10.7759/cureus.93523

**Published:** 2025-09-29

**Authors:** Vivek R Patel, Akshay K Gupta, Jyoti Dwivedi, Nirupma Lal, Afroz Abidi, Aparna Misra

**Affiliations:** 1 Biochemistry, Era's Lucknow Medical College and Hospital, Era University, Lucknow, IND; 2 Biochemistry, Lucknow University, Lucknow, IND; 3 Pathology, Era's Lucknow Medical College and Hospital, Era University, Lucknow, IND; 4 Pharmacology, Era's Lucknow Medical College and Hospital, Lucknow, IND

**Keywords:** anthocephalus indicus, histopathology, metabolic syndrome, streptozotocin, terminalia arjuna

## Abstract

Background: Metabolic syndrome and diabetes mellitus are among the most common chronic disorders worldwide, posing significant challenges to healthcare systems. They are characterized by persistent high blood glucose levels, insulin resistance, and progressive impairment of organs such as the pancreas, liver, kidneys, and heart. In the present study, we can evaluate the protective effects of a combined ethanolic extract of *Anthocephalus indicus* and *Terminalia arjuna* on hematological and histopathological alterations induced by metabolic syndrome in multiple vital organs of rats.

Methods: Male Wistar rats were divided into seven groups (n = 6): normal control, diabetic control (high-fat, high-fructose (HFHF) diet + streptozotocin (STZ)), individual extract-treated, combination-treated, metformin, and atorvastatin-treated. For 16 weeks, all groups (except the normal group) received a high-fat, high-fructose diet, followed by a low-dose streptozotocin (30 mg/kg body weight) injection to induce metabolic syndrome. The treatment phase included administration of individual extracts (500 mg/kg), combination extract (250 + 250 mg/kg), metformin (100 mg/kg), or atorvastatin (20 mg/kg). After the experimental period, animals were sacrificed, and organs, including the pancreas, kidney, liver, heart, lungs, and spleen, were examined histologically using hematoxylin and eosin staining.

Results: The diabetic control group showed significant histopathological damage across all organs, including islet distortion, glomerular hypertrophy, hepatic ballooning, myocardial degeneration, alveolar disruption, and splenic architectural loss. Treatment with individual extracts showed partial protection. However, the combination-treated group exhibited marked histological restoration comparable to metformin, indicating synergistic organ-protective effects. Atorvastatin showed moderate structural recovery in most tissues. Hematological analysis further revealed anemia, leukocytosis, and thrombocytosis in the diabetic control group, while the combination extract significantly improved hemoglobin, red blood cell (RBC), hematocrit (HCT), and normalized leukocyte and platelet counts, effects comparable to metformin.

Conclusion: The combined herbal formulation of *A. indicus* and *T. arjuna* demonstrated significant multiorgan protection in metabolic syndrome-induced rats. These findings suggest the potential of this phytotherapeutic approach as an effective alternative or adjunct in managing metabolic complications.

## Introduction

Metabolic syndrome (MetS) and diabetes mellitus are widespread chronic health conditions that continue to pose major challenges to global healthcare systems. It affects nearly 20-30% of adults worldwide, with prevalence rising to 34% in the United States and about 25-30% in India. Major risk factors include central obesity, insulin resistance, sedentary lifestyle, and dietary patterns. South Asians are particularly vulnerable, developing diabetes and cardiovascular disease at younger ages and lower BMI levels. MetS increases the risk of type 2 diabetes fivefold and cardiovascular events twofold, underscoring the urgent need for effective interventions. These disorders are primarily characterized by persistently elevated blood glucose levels, insulin resistance, and gradual deterioration of essential organs such as the liver, kidneys, pancreas, and heart [1]. Over time, this metabolic imbalance leads to oxidative stress and systemic inflammation, which play a central role in disease progression [2]. At the cellular level, these metabolic disturbances manifest as distinct structural damage across multiple organs. In pancreatic tissue, for instance, the insulin-producing islets of Langerhans often appear shrunken and disrupted. The kidneys typically show glomerular swelling, thickening of the basement membrane, and early signs of nephron loss. Hepatic tissues commonly exhibit fatty changes, ballooning of hepatocytes, and periportal inflammation, features commonly seen in diabetes-related liver injury. Similarly, the myocardium undergoes fibrotic changes, with visible degeneration of cardiac fibers and interstitial edema, contributing to compromised cardiac function [3,4].

In addition to organ-related damage, metabolic syndrome is also accompanied by marked hematological disturbances that add to its systemic complications. Conditions such as anemia, increased leukocyte counts, and abnormal platelet activity are commonly noted in diabetic states, reflecting defective red cell production, heightened inflammatory drive, and enhanced risk of thrombosis. Decline in hemoglobin and hematocrit values is a well-recognized feature of diabetes-related anemia, while rises in leukocyte and platelet counts are indicators of persistent inflammation and a pro-thrombotic environment. These blood-related changes not only act as markers of metabolic imbalance but also actively contribute to the progression of cardiovascular complications such as atherosclerosis, coronary artery disease, myocardial infarction, and stroke, which are major causes of mortality. It also predisposes to renal complications, including diabetic nephropathy, chronic kidney disease, and progressive renal failure. These complications are severe and progressive, highlighting the urgent need for early intervention [5,6]. Among these, plant-based interventions have gained considerable attention due to their multifaceted biological properties and traditional use in chronic disease management. *Anthocephalus indicus* (*A. indicus*) and *Terminalia arjuna (T. arjuna*) are two such medicinal plants with a long-standing reputation in Ayurveda for supporting cardiac, hepatic, renal, and metabolic health [7,8].

Currently, treatment using polyherbal formulations demonstrates enhanced synergistic effects compared to single-herb therapies in managing diabetes mellitus, its associated dyslipidemia, and related complications. The polyherbal approach is considered to offer broader and more effective therapeutic benefits than monotherapy with individual herbs [9-12]. While both plants have demonstrated promising results individually, their combined therapeutic potential, particularly in mitigating diabetes-induced tissue injury, has not been extensively evaluated in experimental models.

The present study was therefore undertaken to assess the protective effects of the combined ethanolic extract of*A. indicus *and *T. arjuna* on hematological and structural changes in vital organs, specifically the pancreas, liver, kidneys, lungs, spleen, and heart in an established rat model of metabolic syndrome.

## Materials and methods

Selection of experimental animals

Adult male albino Wistar rats (180-200 g) were obtained from the Animal House Facility of the Indian Veterinary Research Institute (IVRI), Izatnagar, Bareilly (U.P., India; Reg. No. 1652/PO/Re/S/12/CPCSEA; Certificate No. IAEC/ELMCH/2/22/-1). The animals were kept at the animal house of Era's Lucknow Medical College and Hospital under controlled environmental conditions (23 ± 2 °C, 40-50% relative humidity, 12-hour light/dark cycle). All experimental procedures were conducted by the Committee for the Purpose of Control and Supervision of Experiments on Animals (CPCSEA) and the Institutional Animal Ethics Committee (IAEC) guidelines. Rats were acclimatized for one week before the initiation of the study and were maintained on a high-fat, high-fructose diet with water ad libitum for 16 weeks.

Extraction and standardization procedures for *A. indicus* and *T. arjuna*

A. indicus

One kilogram of dried fruit powder of *A. indicus* was extracted with 10 volumes of 95% ethanol. The mixture was allowed to stand overnight, and the extract was collected the following morning. The residue was re-extracted five times with the same volume of ethanol, and all extracts were pooled. The combined extract was concentrated under reduced pressure (in vacuo) to obtain a solid residue, designated as “Ethanolic Extract.” The dried extract was stored in an airtight container at 4°C until use. The total yield obtained was 103.4 g from 1.0 kg of crude powder. The dried ethanolic extracts were stored in amber airtight containers at 4°C and used within six months of preparation to ensure stability.

*T. arjuna* 

Similarly, 1.0 kg of dried fruit powder of *T. arjuna* was extracted by cold maceration with 10 volumes of 95% ethanol. The mixture was kept overnight, and the supernatant was collected the next morning. The residue was re-extracted five times with the same volume of ethanol, and all extracts were pooled. The combined extract was concentrated using a rotary evaporator (Buchi R-210, Switzerland) under reduced pressure (in vacuo) to dryness. The dried extract, designated as “Ethanolic Extract,” was stored in amber airtight containers at 4°C until further use. The total yield obtained was 87.4 g from 1.0 kg of crude powder. All extracts were used within six months of preparation to ensure stability.

Toxicological/safety assessments

Acute Toxicity of the Combined Formulation (CF) of A. indicus and T. arjuna in Wistar Albino Rats

Previous studies have reported that *A. indicus* and *T. arjuna* are non-toxic at oral doses up to 2000 mg/kg body weight in Wistar rats, with no observed mortality or significant behavioral or physiological changes [13,14]. An acute toxicity study was conducted using CF (n = 4 per group), which received oral doses of 300, 500, 1000, 1500, and 2000 mg/kg body weight (b.w) following Organisation for Economic Co-operation and Development (OECD) Guideline 423 [15]. The animals were monitored for 14 days for signs of mortality, behavioral changes, body weight alterations, and food/water intake. As no deaths occurred up to the highest tested dose (2000 mg/kg), the LD₅₀ could not be precisely calculated using Karber’s method. However, based on the absence of mortality, the LD₅₀ is considered to be greater than 2000 mg/kg, indicating a favorable acute safety profile of the individual and combined extracts (Table 1).

**Table 1:** Acute oral toxicity test. No mortality or significant adverse effects were observed in any group at any of the tested doses. Acute oral toxicity (LD₅₀ test) of the combined formulation (CF) in Wistar rats. Groups (G.P 1–5) received single oral doses ranging from 300 to 2000 mg/kg.

Groups	Dose (mg/kg)	Mortality (out of five rats)
G.P – 1	300	0/5 (0%)
G.P – 2	500	0/5 (0%)
G.P – 3	1000	0/5 (0%)
G.P – 4	1500	0/5 (0%)
G.P – 5	2000	0/5 (0%)

Induction of metabolic syndrome

Metabolic syndrome was experimentally induced by administering a high-fat, high-fructose (HFHF) diet to rats for 16 weeks. This dietary model mimics human metabolic stress and promotes insulin resistance, hyperglycemia, and dyslipidemia [16]. To enhance metabolic dysfunction, a single intraperitoneal injection of streptozotocin (STZ) at 30 mg/kg body weight was administered after the dietary regimen, inducing partial β-cell dysfunction [17]. Seventy-two hours post-injection, fasting blood glucose was measured, and animals with levels exceeding 250 mg/dL were considered to have developed metabolic syndrome and were selected for further investigation.

*Preparation of High-Fat, High-Fructose* *Diet *(*HFHFD) and Normal Diet*

The HFHFD was freshly prepared in-house following validated formulations. The composition of the HFHFD and normal diet used in the study is shown in Table 2. The HFHFD was enriched with fat, simple sugars, and cholesterol to induce metabolic stress mimicking human metabolic syndrome, whereas the normal diet provided balanced nutrition primarily from complex carbohydrates and proteins.

**Table 2:** Composition of high-fructose high-fat diet (HFHFD) and normal diet. *Supplied by SRL, Mumbai, India. &Mineral blend included (g/kg of diet): calcium hydrogen phosphate (430), potassium chloride (100), sodium chloride (100), magnesium oxide (10.5), magnesium sulfate (50), ferric oxide (3), ferrous sulfate heptahydrate (5), and a trace element mix containing manganese, copper, cobalt, zinc, and iodine (10). The total composition was adjusted to 1000 g. $Bought at a nearby general store. %Vitamins were added to the dry diet at a dosage of (mg/kg): The composition of the vitamin supplement was based on the information provided on the product packaging.

Constituents	Amount in g/Kg diet (HFHFD)	Amount in g/Kg diet (normal)
Fructose/sucrose*	220	120
Fat (Dalda + Vanaspati ghee)^&^	400	50
Casein*	100	168
Maize powder^$^	88	288
Gram powder^$^	60	210
Whole wheat powder^$^	6	216
Jaggery^$^	80	120
Mineral mixture^&^	6	6
Vitamin^%^	10	10
Cholesterol*	20	-
Corn starch*	20	20

Experimental Design and Grouping

In this study, the rats were randomly divided into seven groups, with six animals in each group (Table 3). The goal was to compare the effects of individual plant extracts, their combination, and standard medicines on changes caused by metabolic syndrome. All animals, except those in the normal group, were given a high-fat, high-fructose (HFHF) diet for 16 weeks to create a condition similar to metabolic syndrome seen in humans. After this period, a low-dose of streptozotocin (STZ) was injected to further develop diabetic features. Once the disease condition was confirmed, treatment began. Some groups received extracts of either *A. indicus* or *T. arjuna*, while another group received a mix of both in lower doses. Two groups were treated with standard medicines, one with metformin and another with atorvastatin. All treatments were given orally for 21 days. The normal and HFHF diets were specially prepared by the researchers to maintain consistency.

**Table 3:** Presents a detailed overview of the experimental grouping, including dietary protocols, STZ administration status, treatment regimens, corresponding dosages, and routes of administration for each group. HFHF: high-fat high-fructose diet; STZ: streptozotocin. Note: Both the normal and HFHF diets were custom-prepared using validated formulations.

Group	Group name	Diet	STZ injection (30 mg/kg, i.p.)	Treatment	Dose (mg/kg b.w.)	Route
I	Normal control	Normal diet	No	None	-	-
II	Diabetic control	HFHF diet	Yes	None	-	-
III	*A. indicus *treated	HFHF diet	Yes	*A. indicus* extract	500	Oral
IV	*T. arjuna* treated	HFHF diet	Yes	*T. arjuna* extract	500	Oral
V	Combination treated	HFHF diet	Yes	*A. indicus* + *T. arjuna*	250 + 250	Oral
VI	Metformin treated	HFHF diet	Yes	Metformin	100	Oral
VII	Atorvastatin treated	HFHF diet	Yes	Atorvastatin	20	Oral

Histopathological examination

At the end of the study, all animals were gently and humanely euthanized with chloroform in a closed chamber until adequate anesthesia was achieved prior to sample collection to avoid any pain or distress. Immediately afterward, important organs such as the liver, kidneys, pancreas, and heart were carefully removed. Each organ was rinsed with chilled normal saline to clear out any remaining blood and ensure the tissues were clean. They were then placed in 10% neutral buffered formalin and left for at least 48 hours to allow proper fixation. Once fixed, the tissues were processed using standard paraffin embedding techniques. Thin sections, around five micrometers thick, were cut using a rotary microtome and mounted on clean glass slides. The slides were then deparaffinized, rehydrated, and stained with hematoxylin and eosin (H&E), a commonly used stain for general tissue examination [18,19].

The stained slides were observed under a light microscope at both 10x and 40x magnification to examine any structural or pathological changes. High-resolution images were captured using a digital camera attached to the microscope, which were later used for documentation and comparison between different treatment groups.

Statistical analysis

The sample size was determined based on previous experimental studies on metabolic syndrome models, with six rats per group (n = 6), providing sufficient power (80%) at a 5% significance level to detect meaningful differences in biochemical parameters. All results are expressed as mean ± standard error of the mean (SEM). The data were analyzed using one-way analysis of variance (ANOVA) to compare differences among experimental groups, followed by Dunnett's multiple comparison post-hoc test to compare each treatment group with the diabetic control. A p-value of less than 0.05 was considered statistically significant. Statistical analysis was performed using GraphPad Prism software (version 5, GraphPad Software Inc., San Diego, CA, USA).

## Results

Histopathological observations

Pancreas

**Figure 1:**
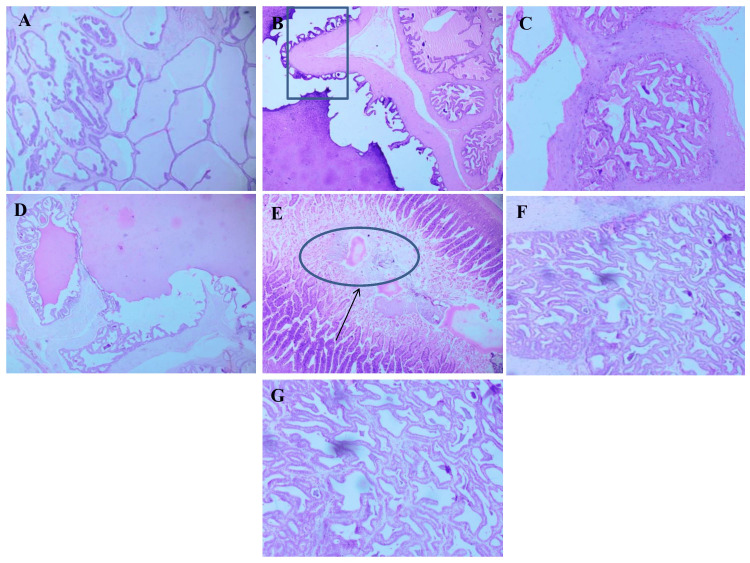
Comparative histopathology of pancreas in normal, diabetic, and drug-treated groups illustrating islet integrity and inflammation. Representative photomicrographs of pancreatic tissue sections from each experimental group stained with H&E, 10× magnification. (A) Normal control showing intact islets of Langerhans. (B) Diabetic control with distorted, necrotic islets. (C) The *A. indicus*-treated group showed partial structural recovery. (D) The *T. arjuna*-treated group with moderate restoration. (E) Combination-treated group exhibited marked preservation of islet architecture. (F) Metformin-treated group showed near-normal pancreatic morphology. (G) Atorvastatin-treated group with mild-to-moderate histological improvement.

Histological analysis of the pancreas revealed clear and consistent morphological differences among the experimental groups. In Figure 1, the normal control group, the pancreatic sections showed healthy islets of Langerhans with well-defined borders, uniform cellularity, and intact acinar tissue. The diabetic control group, however, exhibited significant degeneration, characterized by shrunken and distorted islets, prominent cellular vacuolization, pyknotic nuclei, and marked inflammatory infiltration. Rats treated with *A. indicus *and *T. arjuna* showed partial restoration, where the islet cells appeared relatively organized with moderate reduction in vacuolation and inflammation. The group receiving the combined formulation exhibited better preservation of islet architecture with larger, more compact islets, reduced necrotic changes, and minimal inflammatory response. In the metformin-treated group, the pancreatic histology resembled that of the normal control, with well-preserved islets and acinar structures. The atorvastatin-treated group showed mild-to-moderate recovery, with partially reorganized islets and a reduction in pathological features.

Kidney

**Figure 2:**
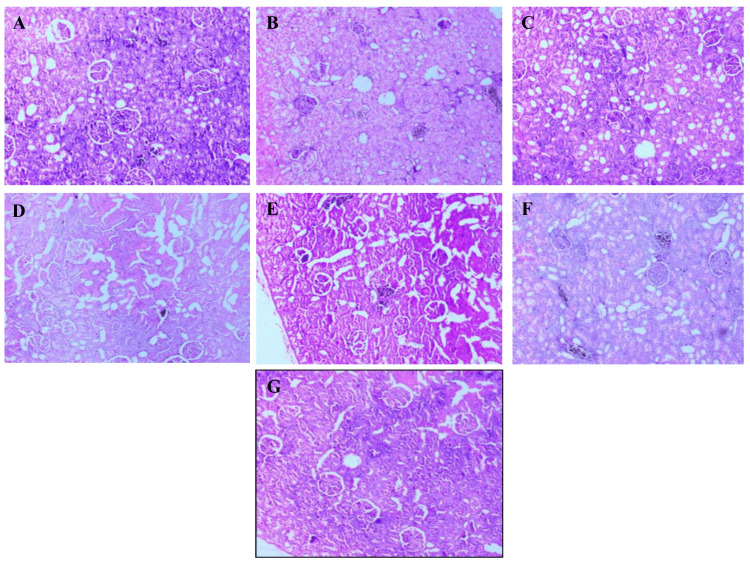
Representative histological sections of the kidney from different experimental groups. Representative photomicrographs of H&E-stained kidney tissue sections from experimental groups (10× magnification): (A) Normal control – intact glomeruli with well-defined Bowman's capsules and normal tubular epithelium. (B) Diabetic control – glomerular hypertrophy, narrowed Bowman's space, tubular dilation, and inflammatory infiltration. (C) *A. indicus*-treated group – partial restoration of glomerular and tubular structure. (D) *T. arjuna*-treated group – moderate improvement with reduced congestion. (E) Combination-treated group – near-normal renal histology with minimal inflammation. (F) Metformin-treated group – marked recovery of glomerular and tubular morphology. (G) Atorvastatin-treated group – moderate repair with reduced pathological features.

Microscopic analysis of renal tissues revealed significant pathological variations among the study groups. The normal control group displayed intact renal histology, with clearly defined Bowman's capsules, uniformly structured glomeruli, and normal tubular epithelium. In the diabetic control group, kidneys showed marked glomerular hypertrophy, narrowing of Bowman’s space, tubular dilation, and evidence of cellular degeneration. There were also inflammatory cell infiltration and early signs of interstitial fibrosis. Treatment with *A. indicus *led to partial protection, with modest improvement in glomerular and tubular morphology. The *T. arjuna*-treated group showed similar improvement, with better preservation of nephron structure and reduced congestion. Rats treated with the combined extract exhibited near-normal renal architecture, including well-preserved glomeruli and minimal inflammation, suggesting a synergistic nephroprotective effect. The metformin-treated group showed significant recovery in renal histology, with restoration of glomerular symmetry and tubular structure. Atorvastatin-treated rats demonstrated moderate improvement, with reduced glomerular swelling and partial reversal of tubular damage (Figure 2).

Heart

**Figure 3:**
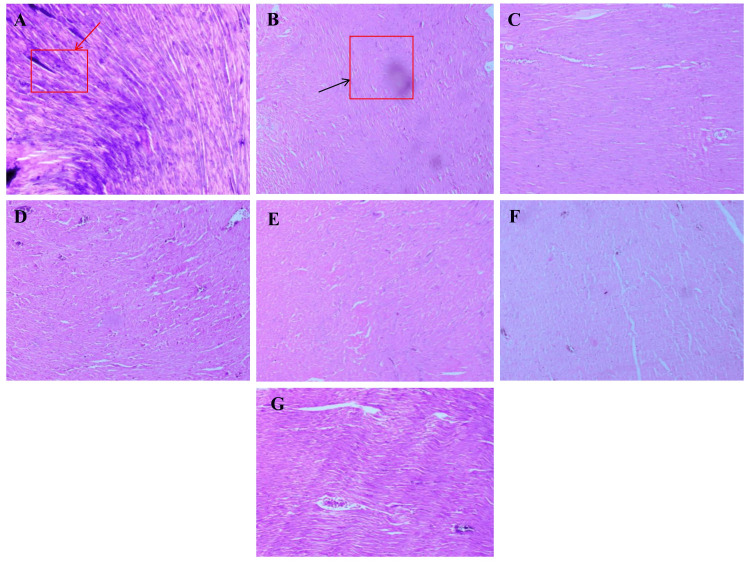
Representative histopathological changes of the heart from different experimental groups. Representative photomicrographs of H&E-stained cardiac tissue sections from different experimental groups (10× magnification): (A) Normal control – well-organized myocardial fibers with distinct striations and central nuclei. (B) Diabetic control – disrupted cardiac architecture with myofibrillar degeneration, interstitial edema, and inflammatory infiltration. (C) *A. indicus*-treated group – partial structural improvement and reduced inflammation. (D) *T. arjuna*-treated group – moderate preservation of myocardial fibers and reduced edema. (E) Combination-treated group – near-normal cardiac morphology indicating synergistic protection. (F) Metformin-treated group – well-preserved myocardial structure similar to the normal group. (G) Atorvastatin-treated group – mild-to-moderate recovery with improved tissue organization.

Cardiac tissue examination revealed prominent histopathological differences among groups. The normal control group showed intact myocardial fibers with striations, centrally located nuclei, and no evidence of inflammation or fibrosis. In contrast, the diabetic control group exhibited severe myocardial disarray, loss of cross striations, myofibrillar degeneration, interstitial edema, and inflammatory cell infiltration. *A. indicus*-treated rats showed partial improvement in myocardial structure, with reduced degeneration and mild reduction in inflammation. *T. arjuna* treatment offered similar cardioprotection, with better preservation of myocardial architecture and less interstitial disruption. The combination-treated group demonstrated near-normal cardiac histology, characterized by aligned myofibers, preserved striations, and minimal pathological changes, indicating a synergistic cardioprotective effect. Metformin-treated rats showed significant reversal of myocardial damage, closely resembling the normal structure. Atorvastatin treatment resulted in moderate recovery, with partial reorganization of muscle fibers and decreased edema (Figure 3).

Spleen

**Figure 4:**
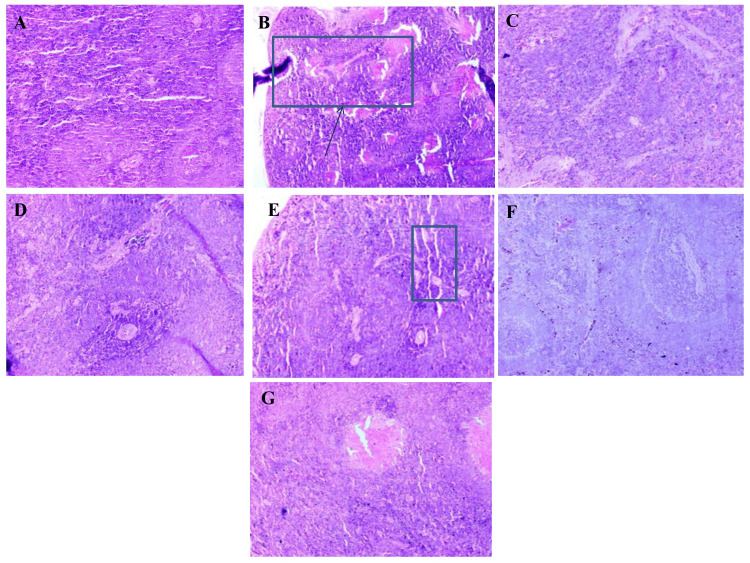
Representative histological sections of spleen from different experimental groups. Representative photomicrographs of H&E-stained spleen tissue sections from experimental groups (10× magnification): (A) Normal control – well-defined white and red pulp with normal lymphoid follicles. (B) Diabetic control – disrupted splenic architecture with reduced white pulp, expanded red pulp, and vascular congestion. (C) *A. indicus*-treated group – partial improvement in pulp structure and reduced congestion. (D) *T. arjuna*-treated group – moderate structural recovery with better pulp definition. (E) Combination-treated group – near-normal splenic architecture with minimal abnormalities. (F) Metformin-treated group – restored splenic tissue closely resembling normal histology. (G) Atorvastatin-treated group – moderate recovery with residual white pulp atrophy and mild vascular changes.

Histological analysis of splenic tissues showed clear structural variations among the experimental groups. The normal control group displayed intact splenic architecture with clearly defined white and red pulp regions, normal lymphoid follicles, and no evidence of congestion or necrosis. In the diabetic control group, marked architectural disruption was observed, characterized by reduced white pulp, expansion of red pulp, vascular congestion, and mild lymphocytic depletion. Treatment with *A. indicus* and *T. arjuna* individually resulted in partial restoration of splenic structure, with modest improvement in pulp definition and reduced congestion. The combination extract-treated group showed substantial recovery, with near-normal architecture, well-defined white pulp, and minimal histological abnormalities. The metformin-treated rats exhibited prominent histological recovery, closely resembling the normal control. Atorvastatin-treated spleens showed moderate improvement, though white pulp remained slightly atrophic with residual vascular changes (Figure 4).

Liver

**Figure 5:**
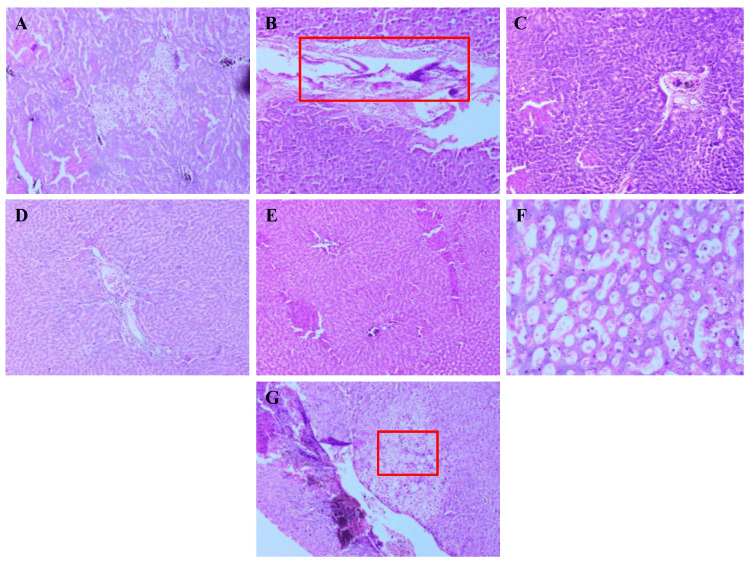
Representative histological sections of liver from different experimental groups. Representative photomicrographs of H&E-stained liver tissue sections from experimental groups (10× magnification): (A) Normal control – well-organized hepatic cords, intact hepatocytes, and prominent central veins. (B) Diabetic control – extensive ballooning degeneration, vacuolated cytoplasm, sinusoidal dilatation, and inflammatory infiltration. (C) *A. indicus-*treated group – partial recovery with reduced vacuolation and improved hepatic arrangement. (D) *T. arjuna*-treated group – moderate improvement with better organization of hepatic architecture. (E) Combination-treated group – near-normal liver histology and minimal inflammatory changes. (F) Metformin-treated group – prominent restoration of hepatic plates and reversal of diabetic lesions. (G) Atorvastatin-treated group – moderate improvement with decreased ballooning and partial structural recovery.

Histological evaluation of liver tissues revealed notable alterations in hepatic architecture across groups. The normal control group exhibited regular hepatic cords, prominent central veins, and well-preserved hepatocytes without any signs of necrosis or inflammation. In contrast, the diabetic control group showed extensive hepatic damage, including ballooning degeneration, cytoplasmic vacuolation, sinusoidal dilatation, and marked inflammatory infiltration around the portal and central vein regions. Treatment with *A. indicus* and *T. arjuna* individually led to partial restoration of liver histology, reflected by reduced vacuolation and better organization of hepatic cords, though some inflammatory changes persisted. The combination-treated group exhibited substantial hepatoprotective effects, with near-normal tissue architecture and minimal histological abnormalities. Metformin-treated animals displayed prominent recovery with organized hepatic plates and almost complete reversal of diabetic lesions. Atorvastatin-treated rats showed moderate improvement, with decreased ballooning and partial restoration of normal hepatic structure (Figure 5).

Lungs

**Figure 6:**
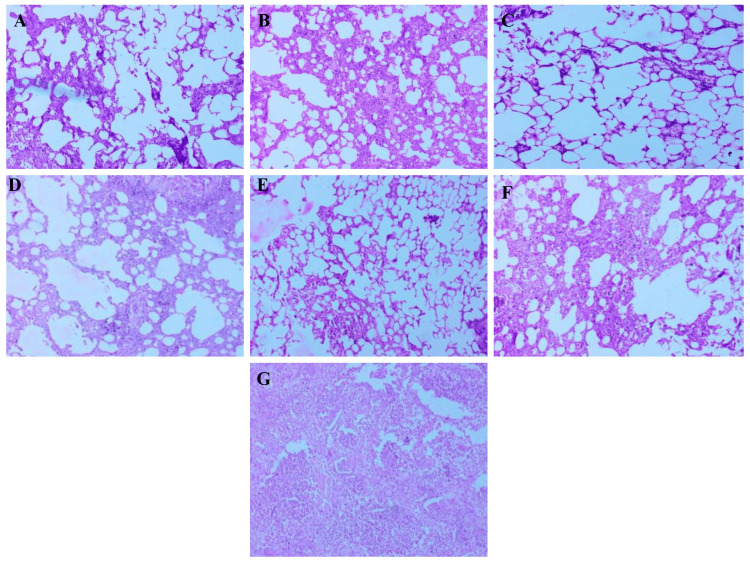
Representative histological sections of lungs from different experimental groups. Representative photomicrographs of H&E-stained lung tissue sections from experimental groups (10× magnification): (A) Normal control – preserved pulmonary architecture with clear alveolar spaces and thin septa. (B) Diabetic control – alveolar septal thickening, vascular congestion, and marked inflammatory infiltration. (C) *A. indicus*-treated group – partial recovery with reduced congestion and improved alveolar structure. (D) *T. arjuna*-treated group – moderate restoration of alveolar walls and septal thinning. (E) Combination-treated group – near-normal lung architecture with minimal inflammation. (F) Metformin-treated group – well-preserved alveolar integrity and negligible inflammatory changes. (G) Atorvastatin-treated group – moderate structural recovery with mild septal thickening.

Histological examination of lung tissues demonstrated distinct pathological differences across groups. The normal control group exhibited intact pulmonary architecture with clear alveolar spaces, thin septa, and no signs of inflammation. The diabetic control group displayed substantial structural disruption, including alveolar septal thickening, vascular congestion, and marked inflammatory infiltration, indicating lung injury associated with metabolic syndrome. Treatment with *A. indicus* and *T. arjuna* led to partial recovery, reflected by reduced congestion and partial normalization of alveolar walls. The combination-treated group exhibited significant improvement, with well-preserved alveolar integrity, minimal septal thickening, and nearly normal histoarchitecture. Metformin-treated lungs showed near-complete restoration, with clear septal margins and negligible inflammatory features. Atorvastatin-treated rats showed moderate recovery, with residual septal thickening and mild inflammatory changes (Figure 6).

Interpretation of hematological parameters

Hemoglobin (Hb)

**Figure 7:**
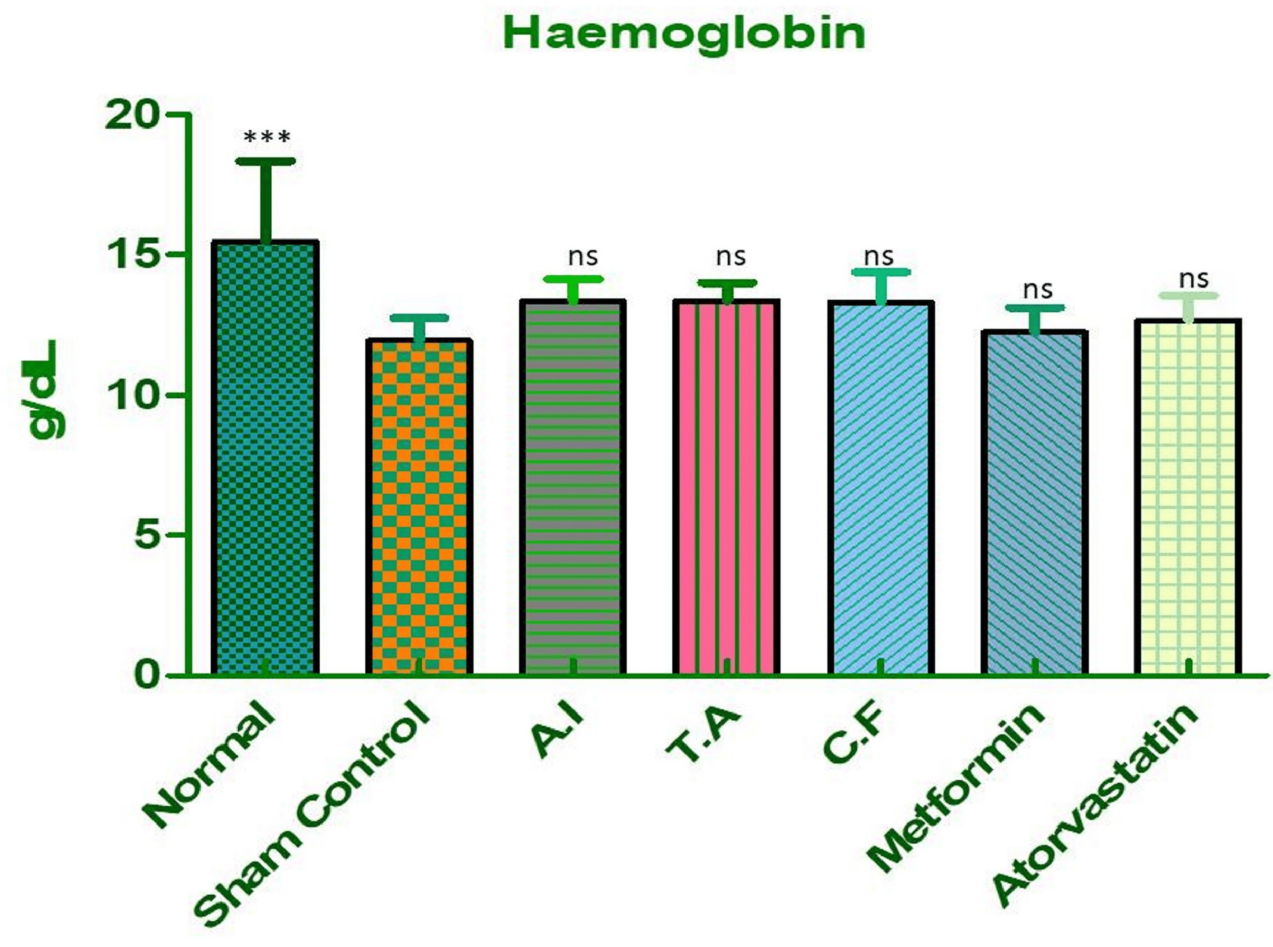
Hemoglobin levels in different experimental groups of Wistar rats. Effect of *A. indicus* (AI), *T. arjuna* (TA), their combination (CF), and standard drugs (metformin, atorvastatin) on Hb levels in Wistar rats. A marked fall in Hb was observed in the sham control compared to normal (p < 0.001), indicating anemia. Treatment with AI, TA, CF, metformin, and atorvastatin showed a trend towards improvement, but differences vs. sham control were statistically non-significant. Data are expressed as mean ± SEM (n = 6).

In the normal control group, hemoglobin levels remained within the expected physiological range, indicating healthy erythropoiesis and stable oxygen transport. However, rats exposed to an HFHF diet followed by STZ injection (sham control group) showed a marked reduction in hemoglobin concentration. This decline is characteristic of anemia associated with metabolic syndrome and is likely linked to chronic inflammation and oxidative stress. Treatment with either *A. indicus* or *T. arjuna* resulted in modest improvements in hemoglobin levels. Notably, the group receiving the CF extract exhibited a substantial increase, with hemoglobin values nearly matching those observed in the metformin-treated group. This suggests a synergistic effect when both plant extracts are used together, possibly enhancing red cell production and reducing oxidative damage, as shown in Figure 7 and Table 4.

**Table 4:** Dunnett's multiple comparison test for hemoglobin levels in different experimental groups. Dunnett's multiple comparison test comparing Hb levels among groups. Sham control vs. normal showed a highly significant decrease (p < 0.001), while all treatment groups (AI, TA, CF, metformin, atorvastatin) showed non-significant changes (ns) when compared with sham control. Values are mean ± S.E. of six rats. AI: *A. indicus*, TA: *T. arjuna*, CF: combined formulation. Statistical significance *P < 0.05, **P < 0.01, ***P < 0.001.

Dunnett's multiple comparison test	Mean difference	Q	Significant? P < 0.05?	Summary	95% CI of diff
Sham control vs normal	-3.525	5.910	Yes	***	-5.190 to -1.860
Sham control vs AI	-1.400	2.347	No	ns	-3.065 to 0.2649
Sham control vs TA	-1.400	2.347	No	ns	-3.065 to 0.2649
Sham control vs CF	-1.375	2.305	No	ns	-3.040 to 0.2899
Sham control vs metformin	-0.3000	0.5030	No	ns	-1.965 to 1.365
Sham control vs atorvastatin	-0.7250	1.215	No	ns	-2.390 to 0.9399

Total Leukocyte Count (TLC)

**Figure 8:**
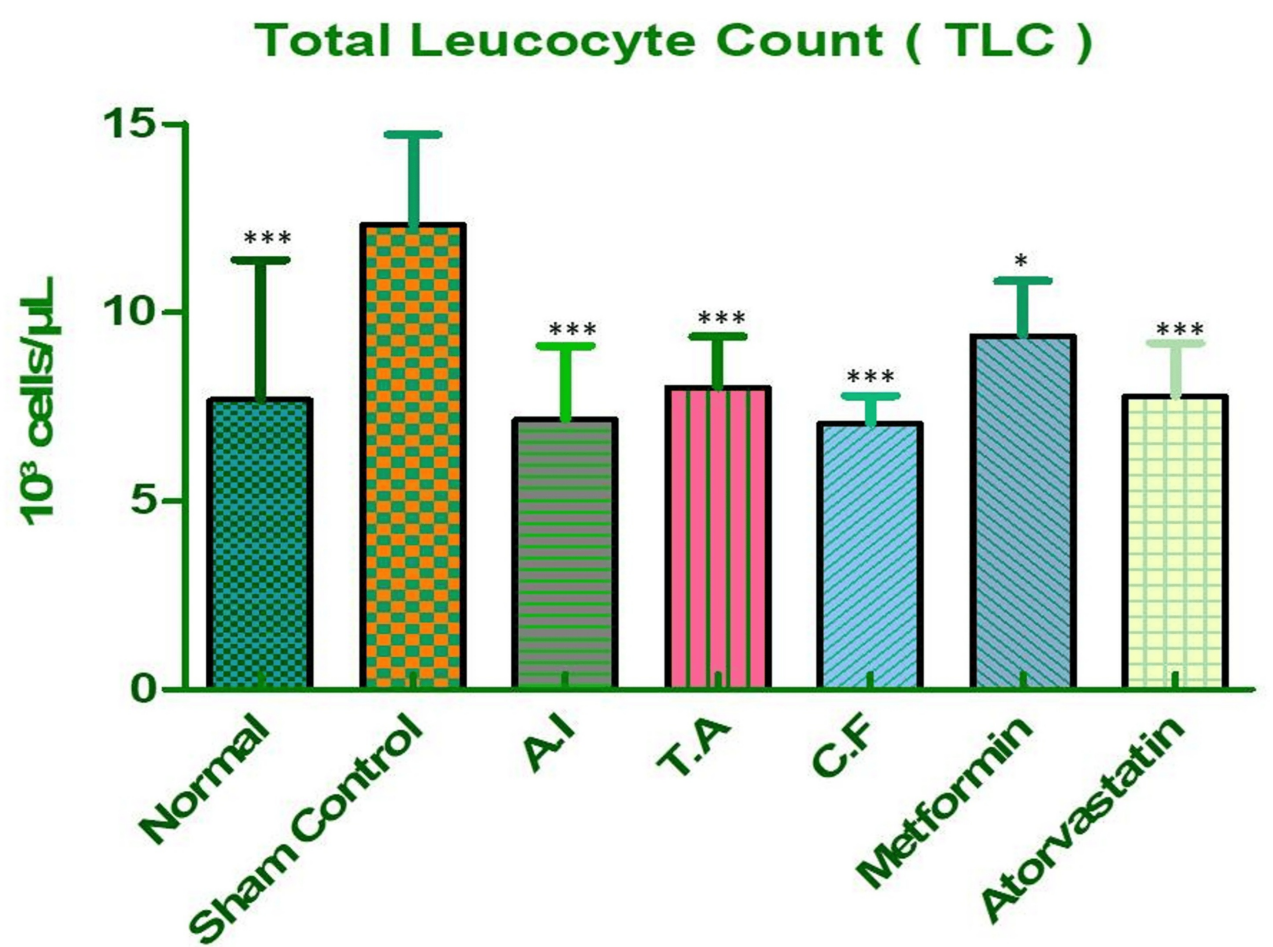
Total leukocyte count (TLC) in different experimental groups of Wistar rats. Effect of AI, TA, their combined formulation (CF), and standard drugs (metformin, atorvastatin) on TLC levels in Wistar rats. Sham control group showed a significant rise in leukocyte count compared to normal (p < 0.001), reflecting systemic inflammation. All treatment groups (AI, TA, CF, metformin, atorvastatin) demonstrated significant reduction in TLC vs. sham control, with CF showing normalization close to metformin. Data are expressed as mean ± SEM (n = 6). AI: *A. indicus*, TA: *T. arjuna.*

Leukocyte counts were significantly elevated in the sham control group, reflecting systemic inflammation induced by metabolic dysregulation. Treatment with individual herbal extracts produced partial reductions in TLC, as shown in Figure 8 and Table 5. However, the combined formulation led to a marked normalization of leukocyte levels, closely resembling the anti-inflammatory profile seen in the metformin group. These findings highlight the immunomodulatory potential of the polyherbal approach in mitigating inflammation associated with metabolic syndrome.

**Table 5:** Dunnett's multiple comparison test for total leukocyte count (TLC) in different experimental groups. Dunnett's multiple comparison analysis of TLC levels across groups. Sham control vs. normal was highly significant (p < 0.001). All treated groups (AI, TA, CF, atorvastatin) showed significant decrease (p < 0.001), while metformin showed a moderate but significant reduction (p < 0.05) vs. sham control. Values are mean ± S.E. of six rats. Statistical significance *P < 0.05, **P < 0.01, ***P < 0.001.

Dunnett's multiple comparison test	Mean diff.	Q	Significant? P < 0.05?	Summary	95% CI of diff
Sham control vs normal	4.650	5.079	Yes	***	2.094-7.206
Sham control vs AI	5.165	5.641	Yes	***	2.609-7.721
Sham control vs TA	4.313	4.710	Yes	***	1.757-6.868
Sham control vs CF	5.275	5.762	Yes	***	2.719-7.831
Sham control vs metformin	2.950	3.222	Yes	*	0.3945-5.506
Sham control vs atorvastatin	4.550	4.970	Yes	***	1.994-7.106

Platelet Count

**Figure 9:**
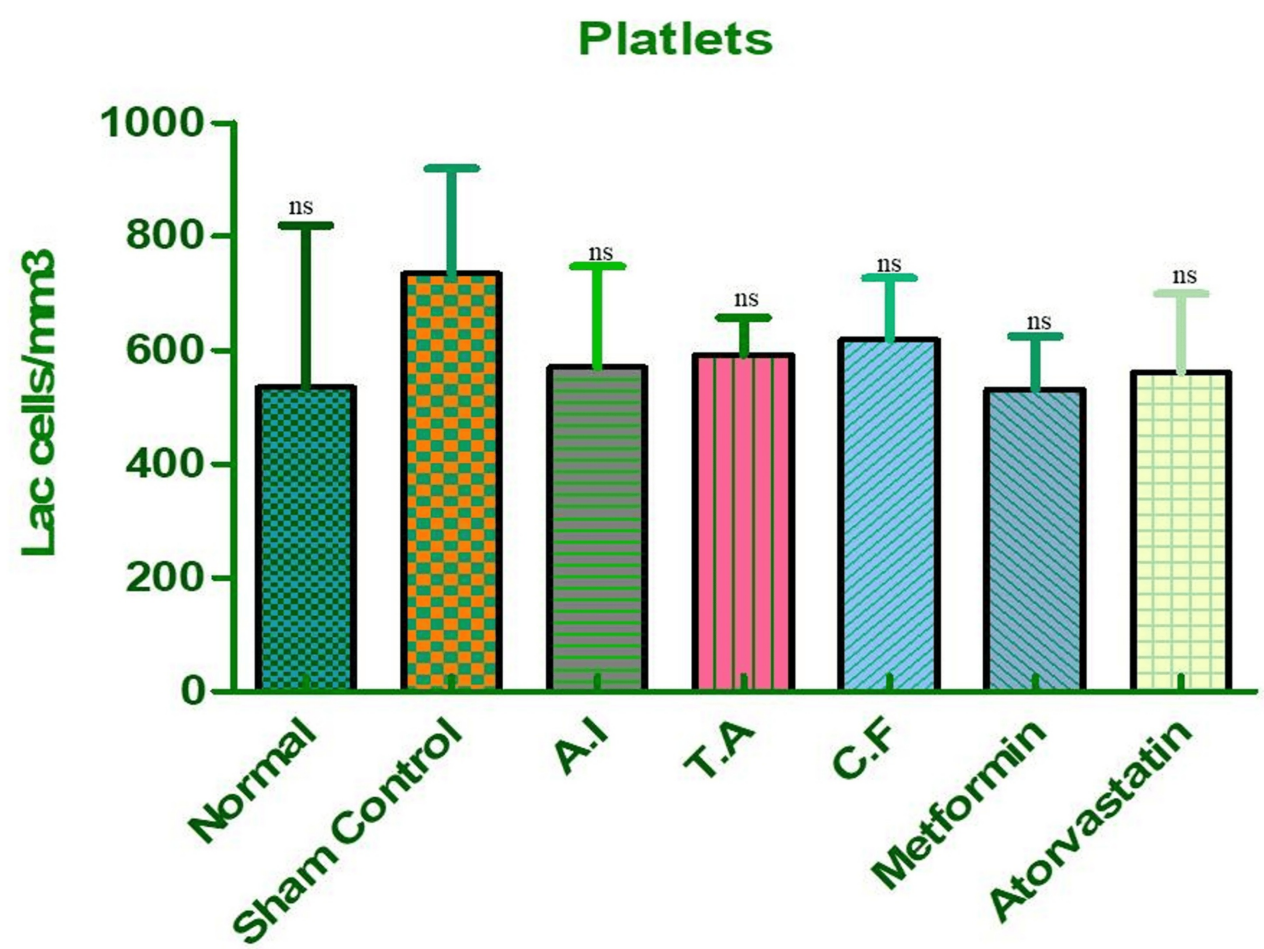
Platelet counts in different experimental groups of Wistar rats. Effect of AI, TA, their combined formulation (CF), and standard drugs (metformin, atorvastatin) on platelet levels in Wistar rats. Sham control rats showed elevated platelet counts, indicating a pro-thrombotic and inflammatory state. Treatment with AI, TA, and CF produced a downward trend, with the combined extract (CF) reducing platelet counts closer to normal and comparable to metformin. Atorvastatin also showed a mild reduction. Data are expressed as mean ± SEM (n = 6). AI: *A. indicus*, TA: *T. arjuna*.

Platelet counts were also increased in the sham group, which may reflect enhanced thrombotic risk and endothelial dysfunction, both of which are common in metabolic syndrome, as shown in Figure 9 and Table 6. Treatment with the combined extract effectively reduced platelet counts to safer levels, closely aligning with the metformin group. This indicates a possible role in improving vascular health and preventing pro-thrombotic complications.

**Table 6:** Dunnett's multiple comparison test for platelet counts in different experimental groups. Dunnett's multiple comparison for platelet counts across groups. Sham control vs. normal showed an increase but did not reach statistical significance (ns). Similarly, comparisons of AI, TA, CF, metformin, and atorvastatin vs. sham control were also statistically non-significant, though values trended toward reduction. AI: *A. indicus*, TA: *T. arjuna*, CF: combined formulation.

Dunnett's multiple comparison test	Mean diff.	Q	Significant? P < 0.05?	Summary	95% CI of diff
Sham control vs normal	200.0	2.729	No	ns	-4.539 to 404.5
Sham control vs AI	164.8	2.248	No	ns	-39.79 to 369.3
Sham control vs TA	142.5	1.945	No	ns	-62.04 to 347.0
Sham control vs CF	115.5	1.576	No	ns	-89.04 to 320.0
Sham control vs metformin	203.0	2.770	No	ns	-1.539 to 407.5
Sham control vs atorvastatin	174.0	2.375	No	ns	-30.54 to 378.5

Red Blood Cells (RBCs)

**Figure 10:**
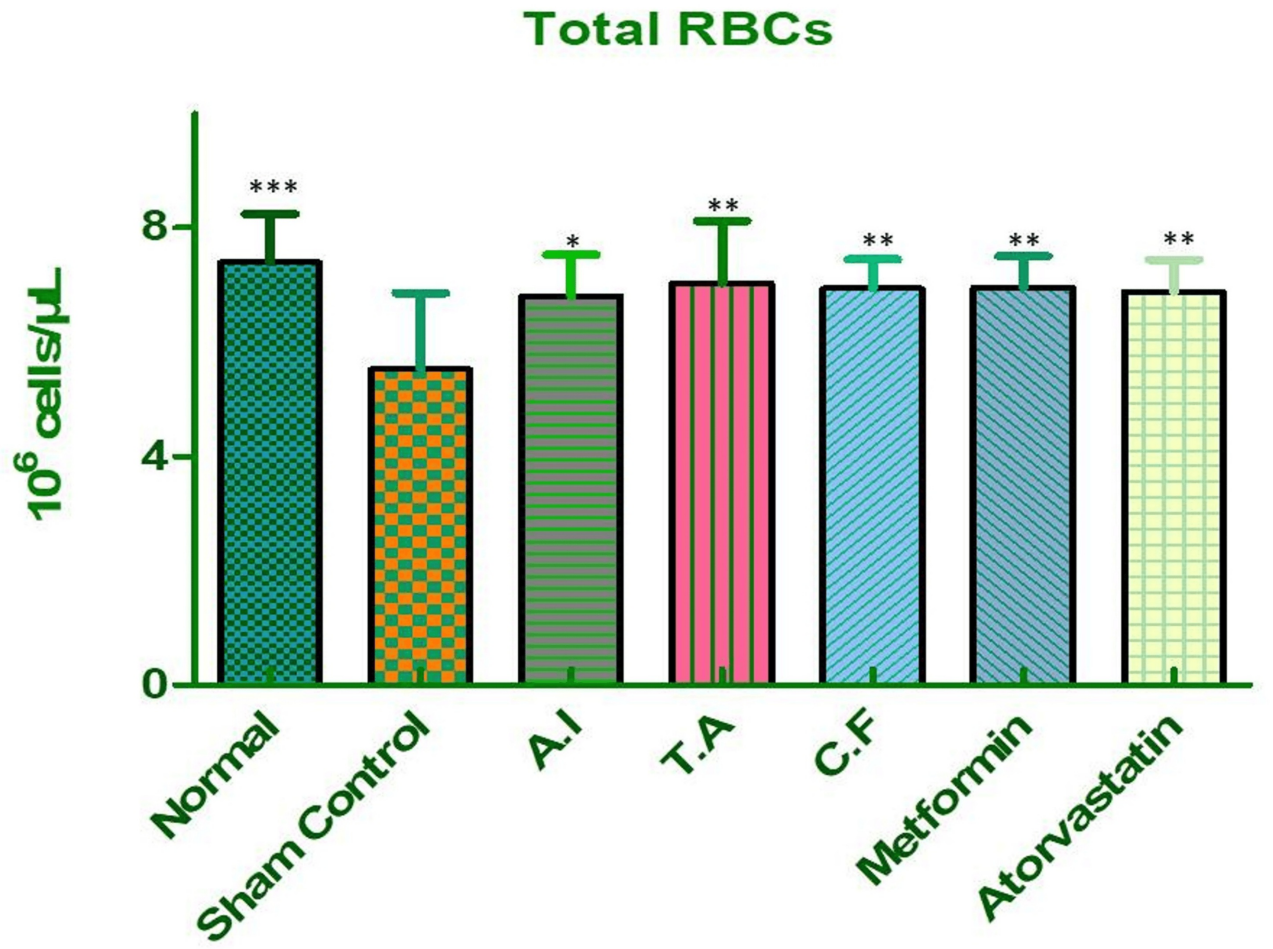
Red blood cell (RBC) count in different experimental groups of Wistar rats. Effect of AI, TA, their combined formulation (CF), and standard drugs (metformin, atorvastatin) on red blood cell levels in Wistar rats. Sham control rats showed a marked reduction in RBCs (p < 0.001 vs. normal), indicating anemia under metabolic stress. Treatment with AI, TA, and CF significantly improved RBC levels compared to sham control, with the combined extract showing the strongest recovery, nearly matching metformin. Atorvastatin also demonstrated significant improvement. Data are expressed as mean ± SEM (n = 6). AI: *A. indicus*, TA: *T. arjuna*.

A decline in RBC count was observed in the sham control group, potentially due to impaired erythropoiesis and increased red cell turnover under metabolic stress. While treatment with individual extracts led to slight improvements, the combination therapy significantly restored RBC levels, achieving results comparable to those of metformin, as shown in Figure 10 and Table 7. This restoration supports the potential of the formulation to correct metabolic anemia and promote red cell stability.

**Table 7:** Dunnett's multiple comparison test for red blood cell (RBC) counts in different experimental groups. Dunnett's multiple comparison test revealed significant reductions in RBC count in sham control vs. normal (p < 0.001). Treatment groups (AI: p < 0.05, TA: p < 0.01, CF: p < 0.01, metformin: p < 0.01, atorvastatin: p < 0.01) all showed significant restoration compared to sham control. Values are mean ± S.E. of six rats. Statistical significance *P < 0.05, **P < 0.01, ***P < 0.001. AI: *A. indicus*, TA: *T. arjuna*, CF: combined formulation.

Dunnett's multiple comparison test	Mean diff.	Q	Significant? P < 0.05?	Summary	95% CI of diff
Sham control vs normal	-1.870	4.953	Yes	***	-2.924 to -0.8163
Sham control vs AI	-1.267	3.358	Yes	*	-2.321 to -0.2138
Sham control vs TA	-1.497	3.967	Yes	**	-2.551 to -0.4438
Sham control vs CF	-1.413	3.742	Yes	**	-2.466 to -0.3588
Sham control vs metformin	-1.430	3.788	Yes	**	-2.484 to -0.3763
Sham control vs atorvastatin	-1.350	3.576	Yes	**	-2.404 to -0.2963

Hematocrit (HCT)

**Figure 11:**
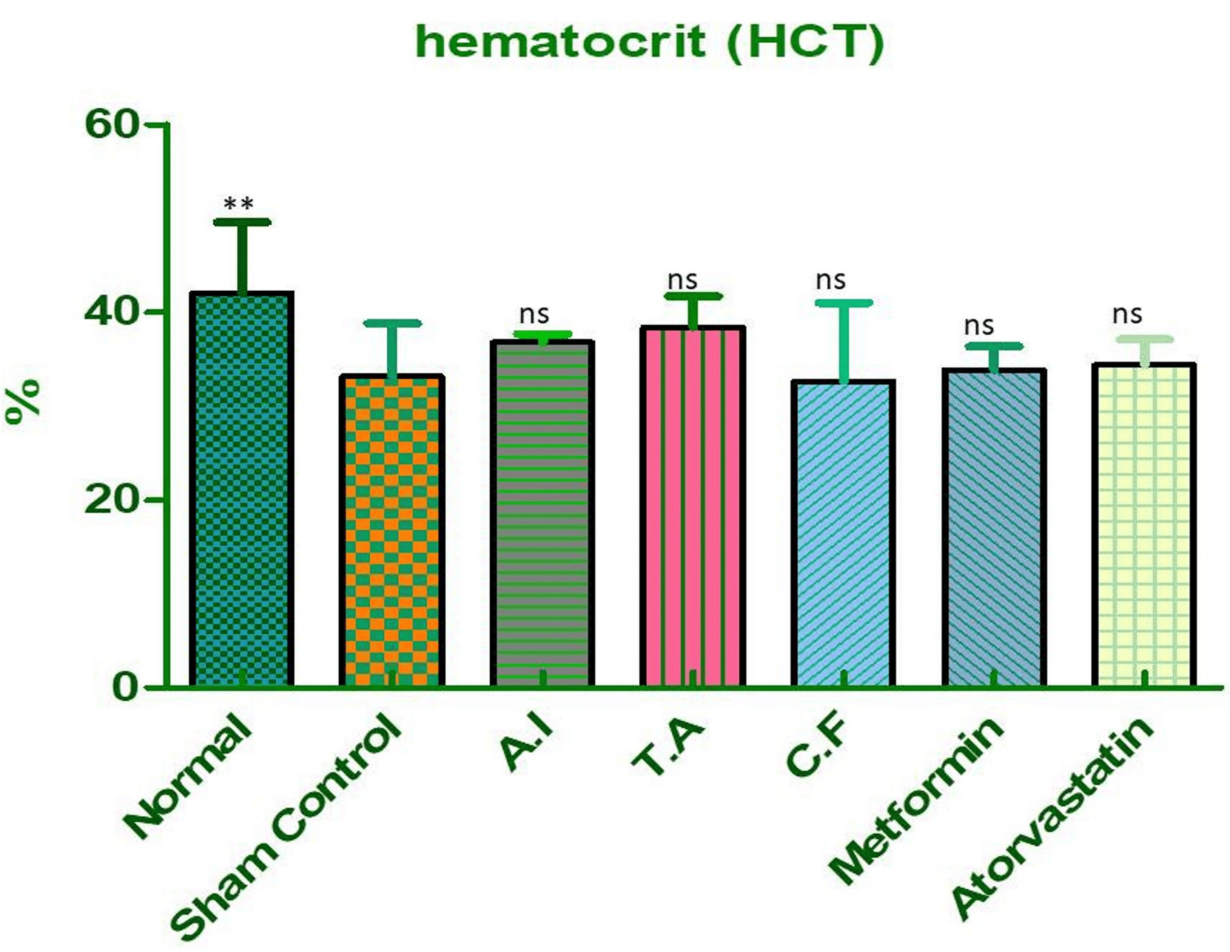
Hematocrit (HCT) percentage in different experimental groups of Wistar rats. Effect of AI, TA, their combined formulation (CF), and standard drugs (metformin, atorvastatin) on hematocrit levels in Wistar rats. Sham control group showed a significant fall in HCT (p < 0.01 vs. normal), reflecting reduced oxygen-carrying capacity and anemia associated with metabolic syndrome. Treatment with AI and TA showed mild, non-significant improvements. The combined extract (CF) restored HCT values close to normal and was comparable to metformin. Atorvastatin also showed a mild, non-significant effect. Data are expressed as mean ± SEM (n = 6). AI: *A. indicus*, TA: *T. arjuna*.

A significant drop in hematocrit levels was also noted in the sham control group, reflecting decreased packed cell volume and reduced oxygen-carrying capacity, a typical feature of anemia associated with metabolic syndrome. Treatment with AI or TA individually resulted in a slight improvement. However, the combined formulation significantly enhanced HCT values, nearly matching those of the metformin group, as shown in Figure 11 and Table 8. This indicates improved erythropoietic function and better hematological recovery, further emphasizing the formulation’s systemic protective role.

**Table 8:** Dunnett's multiple comparison test for hematocrit (HCT) in different experimental groups. Dunnett's multiple comparison analysis showed a significant reduction in HCT in sham control vs. normal (p < 0.01). Comparisons of AI, TA, CF, metformin, and atorvastatin vs. sham control were statistically non-significant (ns), though CF showed the best recovery trend. Values are mean ± S.E. of six rats. Statistical significance *P < 0.05, **P < 0.01, ***P < 0.001. AI: *A. indicus*, TA: *T. arjuna*, CF: combined formulation.

Dunnett's multiple comparison test	Mean diff.	Q	Significant? P < 0.05?	Summary	95% CI of diff
Sham control vs normal	-8.875	3.901	Yes	**	-15.23 to -2.524
Sham control vs AI	-3.650	1.604	No	ns	-10.00 to 2.701
Sham control vs TA	-5.275	2.318	No	ns	-11.63 to 1.076
Sham control vs CF	0.4750	0.2088	No	ns	-5.876 to 6.826
Sham control vs metformin	-0.6750	0.2967	No	ns	-7.026 to 5.676
Sham control vs atorvastatin	-1.225	0.5384	No	ns	-7.576 to 5.126

The combined administration of *A. indicus *and *T. arjuna* demonstrated notable hematological benefits in rats with HFHF-STZ-induced metabolic syndrome. It improved hemoglobin concentration, RBC and HCT levels, and reduced leukocytosis and thrombocytosis, all of which are key indicators of systemic metabolic health. These effects were comparable to those achieved by metformin, suggesting that the polyherbal formulation may serve as a safe and natural alternative for managing hematological complications in metabolic syndrome.

## Discussion

The present study investigated the histopathological and haematological effects of a combined formulation of *A. indicus* and *T. arjuna* on multiple organs affected by metabolic syndrome. Chronic exposure to a HFHF diet followed by streptozotocin administration is a well-established model for inducing insulin resistance, oxidative stress, and low-grade systemic inflammation, mimicking human metabolic syndrome conditions [20]. In the pancreas, diabetic rats showed marked shrinkage and vacuolation of the islets of Langerhans, consistent with β-cell injury and functional decline due to persistent hyperglycemia and oxidative insult [21]. Treatment with either plant extract offered partial protection, but their combination notably restored islet architecture, indicating synergistic cytoprotective potential. Similar pancreatic preservation was observed in the metformin group, reinforcing the formulation’s therapeutic relevance. Kidney sections of diabetic rats showed classical signs of nephropathy, including glomerular hypertrophy, tubular degeneration, and interstitial inflammation. These features align with diabetic-induced hyperfiltration, glomerulosclerosis, and oxidative stress-mediated renal damage [22]. Both *A. indicus* and *T. arjuna* demonstrated mild nephroprotection, while their combination substantially reversed structural damage comparable to metformin, suggesting improved anti-oxidative and anti-inflammatory activity [23]. In cardiac tissues, diabetes-induced myofibrillar degeneration, interstitial edema, and loss of striations are hallmarks of diabetic cardiomyopathy [24]. The protective role of *T. arjuna* in maintaining myocardial structure is well documented, attributed largely to its flavonoids and saponins that possess free radical scavenging and vasodilatory effects [25]. The combined extract restored near-normal architecture, paralleling Metformin's cardioprotective effect. Liver histology in diabetic rats revealed ballooning degeneration, necrosis, and inflammatory infiltration, common features of non-alcoholic fatty liver disease (NAFLD) linked to metabolic syndrome [26]. Both individual and combined extract treatment improved hepatic organization, with the combined group showing the most pronounced improvement. These effects could be attributed to the hepatoprotective nature of phytoconstituents such as betulinic acid, tannins, and phenolics, which are known in both plants [27]. Spleen tissue damage in diabetic rats, evidenced by white pulp shrinkage and red pulp expansion, suggests immune dysregulation and chronic systemic inflammation [28]. Restoration of splenic architecture in treated groups, especially the combination group, indicates anti-inflammatory effects at the lymphoid level, possibly by modulating cytokine response. Pulmonary changes, including alveolar septal thickening and inflammatory infiltration, in the diabetic group confirm lung involvement in metabolic syndrome, which is increasingly recognized in recent studies [29]. The reversal of structural damage by the combination extract suggests potential in mitigating oxidative and inflammatory lung injury, a relatively unexplored yet critical area in metabolic dysfunction.

In addition to organ-level recovery, the CF also produced marked improvements in hematological parameters. Diabetic animals in the sham group showed reduced hemoglobin, red blood cell count, and hematocrit, indicating anemia commonly associated with metabolic stress. Moreover, elevated leukocyte and platelet counts reflected systemic inflammation and a pro-thrombotic state. Treatment with *A. indicus* and *T. arjuna* individually provided partial improvements, but their combination significantly restored hematological balance. Hemoglobin, RBC, and HCT levels improved significantly, and leukocyte and platelet counts were brought closer to normal. These results highlight the CF to promote erythropoiesis and suppress inflammation, further supported by comparable outcomes in the metformin group [30].

Altogether, the CF exhibited broad-spectrum efficacy, protecting both tissue architecture and hematological homeostasis in a model of metabolic syndrome. Its overall effect was similar to that of metformin across multiple organ systems and systemic markers. Future investigations focusing on molecular pathways, and long-term safety assessments will be vital for establishing its clinical applicability and translational potential.

Limitations

This study has certain limitations. The sample size was relatively small, and the duration of treatment was limited to 16 weeks, which may not fully reflect long-term outcomes. As this was an animal model, the findings cannot be directly extrapolated to humans without further validation in clinical studies. In addition, while preliminary phytochemical profiling of the extracts was included, detailed molecular pathway analysis and advanced chromatographic data were not explored in this manuscript, as they form part of separate ongoing work. Future studies with larger cohorts, longer duration, and mechanistic evaluations will be important to strengthen these findings.

## Conclusions

The present study shows that the combination of *A. indicus* and *T. arjuna* offers strong protection against the damage caused by metabolic syndrome. When this CF was given to rats with HFHF diet and STZ-induced metabolic stress, it helped restore the normal structure of several important organs, including the pancreas, liver, kidneys, heart, lungs, and spleen. In many cases, the extent of tissue repair was similar to what was seen with metformin, a well-established antidiabetic drug. This suggests that the CF works effectively, likely due to its antioxidant and anti-inflammatory properties. These structural improvements were strongly supported by the blood analysis in this study. The CF not only increased hemoglobin, RBC count, and hematocrit (important for oxygen transport), but also brought high leukocyte and platelet levels closer to normal, which shows its potential to reduce inflammation and improve blood health. Together, these results indicate that the CF not only protect internal organs, but also supports healthy blood composition. Since metabolic syndrome affects multiple systems simultaneously, its management often requires multi-targeted interventions. The consistent protective effects observed with the combined extracts of *A. indicus *and *T. arjuna* highlight the potential of this polyherbal formulation as a safe, natural, and effective option for long-term management.
